# Supplementary material for: The three-dimensional landscape of tumor-associated macrophages in reactive and neoplastic human lymph nodes
Source: PLOS Digit Health. 2026 Feb 13;5(2):e0001227. doi: 10.1371/journal.pdig.0001227 (PMC12904395; doi:10.1371/journal.pdig.0001227)
Supplement: S1 Fig — (a-c) - Boxplots depicting the pathomic prototype of each diagnostic entity based on pathomic profiles. (a) CD163 features, (b) CD68 features, and (c) global features Pathomic prototyping. (PDF) [file pdig.0001227.s001.pdf]

Supporting Information

S1 - Figure

a.

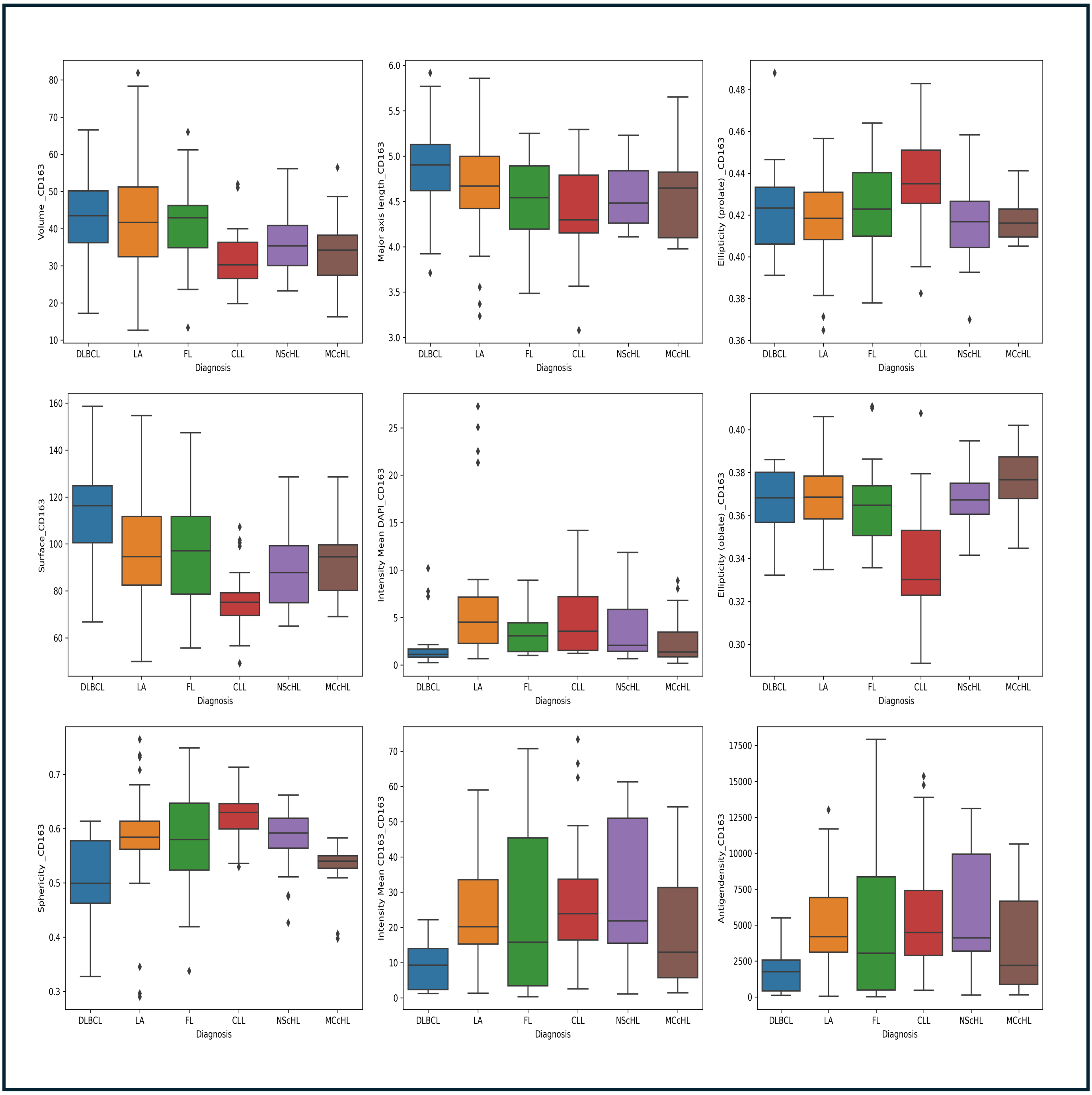

b.

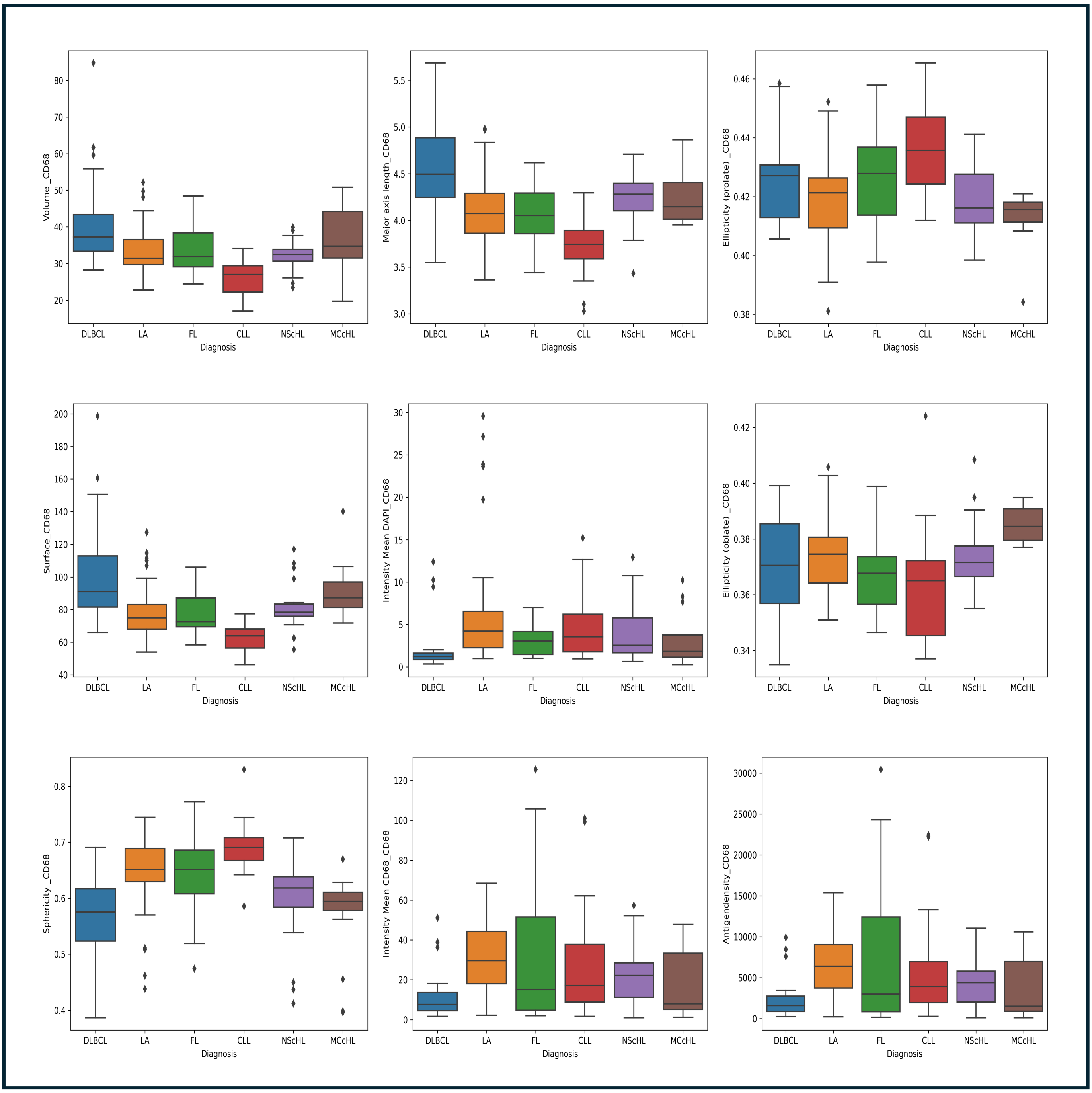
Volume _CD68
Major axis length_CD68
Ellipticity (prolate) _CD68
Surface_CD68
Intensity Mean DAPI_CD68
Ellipticity (oblate) _CD68
Sphericity _CD68
Intensity Mean CD68_CD68
Antigendensity_CD68
DLBCL
LA
FL
CLL
NScHL
MCcHL
Diagnosis

c.

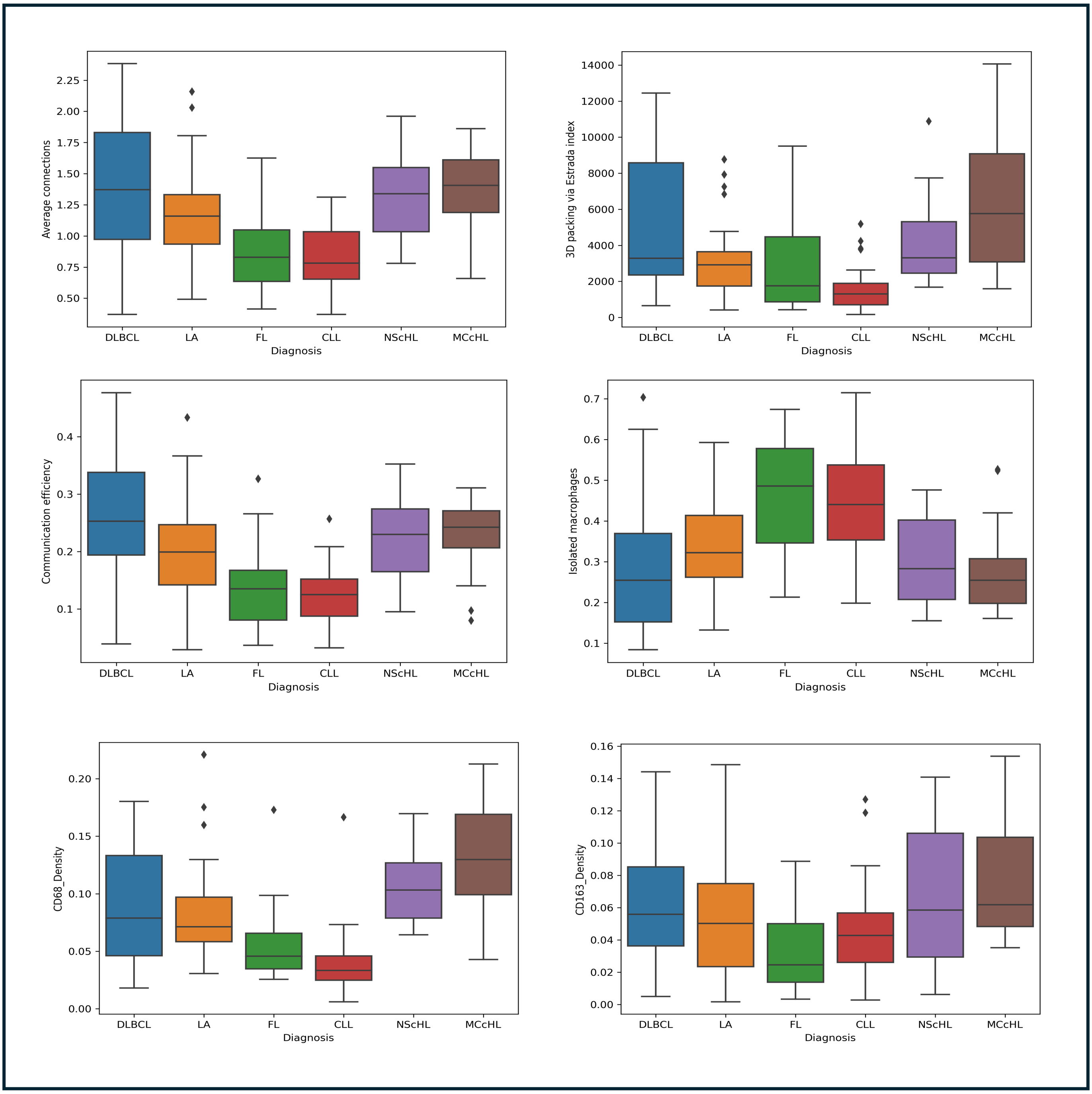

S1_Fig. Boxplots depicting the pathomic prototype of each diagnostic entity based on pathomic profiles. (a) CD 163 features, (b) CD68 features and (c) global features.
