# Supplementary material for: The three-dimensional landscape of tumor-associated macrophages in reactive and neoplastic human lymph nodes
Source: PLOS Digit Health. 2026 Feb 13;5(2):e0001227. doi: 10.1371/journal.pdig.0001227 (PMC12904395; doi:10.1371/journal.pdig.0001227)
Supplement: S2 Fig — All significant changes are highlighted in green. Statistical significance was defined as p < 0,05. (PDF) [file pdig.0001227.s002.pdf]

Supporting Information

S2_Fig. Pathomic prototyping

		CLL	DLBCL	FL	MCcHL	NScHL
Global	CD68 Density	4,15E-07	0,96	0,002	0,001	0,001
Global	CD163 Density	0,43	0,38	0,014	0,049	0,19
Global	Average connections	0,0002	0,06	0,005	0,03	0,039
Global	3D packing via Estrada index	0,0005	0,06	0,24	0,002	0,03
Global	Communication efficiency	0,0002	0,04	0,005	0,13	0,14
Global	Isolated macrophages	0,003	0,08	0,004	0,09	0,19
CD68	Volume	9,08E-06	0,001	0,94	0,25	0,99
CD68	Surface	5,05E-06	0,00009	0,925	0,003	0,2
CD68	Major axis length	0,00006	0,0001	0,79	0,13	0,09
CD68	Sphericity	0,004	0,00002	0,97	0,0001	0,002
CD68	Ellipticity (oblate)	0,014	0,38	0,04	0,00005	0,46
CD68	Ellipticity (prolate)	0,00006	0,076	0,07	0,084	0,93
CD68	Antigen-Density (CD68)	0,18	1,89E-06	0,31	0,01	0,01
CD68	Intensity Mean (CD68)	0,21	8,88E-07	0,23	0,01	0,02
CD68	Intensity Mean (DAPI)	0,47	1,18E-07	0,02	0,01	0,06
CD163	Volume	0,0005	0,75	0,73	0,03	0,02
CD163	Surface	2,94E-06	0,007	0,67	0,34	0,04
CD163	Major axis length	0,044	0,11	0,26	0,55	0,16
CD163	Sphericity	0,008	0,00008	0,64	0,0001	0,75
CD163	Ellipticity (oblate)	5,38E-07	0,65	0,37	0,075	0,98
CD163	Ellipticity (prolate)	0,0006	0,58	0,27	0,82	0,59
CD163	Antigen-Density (CD163)	0,85	1,34E-06	0,27	0,065	0,56
CD163	Intensity Mean (CD163)	0,49	2,66E-06	0,29	0,18	0,38
CD163	Intensity Mean (DAPI)	0,39	4,89E-07	0,023	0,004	0,02

S2_Fig. Pathomic prototyping: Scheme illustrating the pathomic prototype of each diagnostic entity based on the pathomic profiles, corresponding p-values. All significant changes are highlighted in green. Statistical significance was defined as $p < 0,05$.
