# Supplementary material for: The three-dimensional landscape of tumor-associated macrophages in reactive and neoplastic human lymph nodes
Source: PLOS Digit Health. 2026 Feb 13;5(2):e0001227. doi: 10.1371/journal.pdig.0001227 (PMC12904395; doi:10.1371/journal.pdig.0001227)
Supplement: Attachment — Submitted filename: Response to Reviewers.pdf [file pdig.0001227.s005.pdf]

Response to Reviewers

Dear Leo Anthony Celi, dear Iqram Hussain,

thank you for reviewing our manuscript and considering it for publication in PLOS Digital Health. To align with the journal's publication criteria, we recognize the necessity of implementing your suggested revisions. Below, we have provided detailed responses to each comment, and we hope that our revised manuscript now meets the requirements for publication in PLOS Digital Health.

1. Response to Reviewer #1:

    a. A notable deficiency in the manuscript is the absence of microscopy images. Although tri-dimensional reconstructions are presented, no "real" microscopy images are depicted, nor are 3D representations of such images. I propose that examples of each pathology be accompanied by reconstructions.

Answer: Thank you for this valuable comment. We agree that the absence of microscopy images limited the information provided in the manuscript. To address this, we have now included 2D H&E images of all diagnostic entities together with the corresponding 3D CD68/CD163 staining in Figure 4 (p. 13, ll 256-260). In addition, to facilitate comparability, we present conventional 2D thin-section microscopy with CD68 and CD163 staining alongside a 3D CD68/CD163 fluorescence microscopy image in Figure 5 (p. 14, ll. 272-277). We believe these additions substantially improve the visualization and provide deeper insight into 3D microscopy.

Additionally, a pipeline diagram would enhance the explainability of the process.

Answer: Thank you for this helpful suggestion. To improve the clarity of our methodology, we have now included dedicated pipeline diagrams illustrating both the data extraction (Figure 6; p.16, ll. 300-304) and the data analysis steps (Figure 7; p.17, ll. 328-333). These visual representations complement the textual description and are intended to provide readers with a clearer and more intuitive understanding of the data processing workflow. We believe this addition facilitates reproducibility and improves the overall accessibility of our approach.

    b. Regarding segmentations, while IMARIS Advanced Tracking Software is effective, the authors could enhance their workflow by utilizing Machine Learning Tools within the software. These tools offer reduced manual intervention and minimize the likelihood of errors.

Answer: We appreciate this insightful comment. We fully agree that machine learning tools can reduce manual effort and minimize error rates in the analysis of conventional whole-slide images. However, the primary challenge in our dataset lies in its dimensionality—3D versus conventional 2D image data. While state-of-the-art machine learning tools such as *Cellpose* achieve excellent performance on classic 2D datasets, their applicability to complex 3D data remains limited. We therefore believe that, in our setting, established computer vision approaches currently provide a more robust solution than machine learning–based methods.

c. Additionally, object classification pipelines significantly enhance accuracy and improve the separation between classes.

Answer: This is indeed a valid point and an important direction for future work. However, the primary aim of our study was not to maximize classifier performance but rather to analyze and evaluate class-wise classification behavior. Specifically, we sought to assess how distinct the pathomic profiles of TAMs are across different diagnoses (high classification performance) and to identify diagnoses where precise classification is not feasible (low classification performance). In addition, we examined misclassifications to reveal overlaps between diagnoses, thereby providing insights into shared pathomic characteristics. Since the achieved performance significantly outperforms random classification, we consider the interpretation of our results to be justified and extensive classifier optimization beyond the applied level was not required for the present work.

d. The authors briefly mention the statistical analysis, including the number of samples per category and the corresponding p-values for each class. It would be beneficial to include this information in the text for clarity and completeness.

Answer: Thank you for this suggestion. To improve clarity without overloading the main text, we have added Figure 2 to the appendix, which lists all p-values for each comparison together with the corresponding case and image numbers from the Materials and Methods section. We believe this addition enhances transparency while maintaining readability.

e. References should be sequential. In line 53, there's reference [1,29]. Why not [1,2]?

Answer: Thank you for pointing this out. We have revised the manuscript to ensure that all references are listed sequentially, including the correction of [1,29] to [1,2].

2. Reviewer #2:

a. The objective of the study is to explore the relationship of macrophages and the tumors. This relationship isn't sufficiently explored. For example, Figure 2.2's numbers are pretty unimpressive, leading to doubts on whether the model is sufficiently constructed.

Answer: Thank you for pointing out the ambiguity in our manuscript. You are correct that the trained model does not achieve particularly high classification performance, especially for FL, LA, MCcHL, and NScHL. However, this was neither expected nor the primary aim of our analysis. Our objective was to investigate to what extent changes in the pathomic properties of TAMs are diagnosis-specific and where overlaps occur. For this purpose, we optimized the classifier to the level where it performs significantly better than random, thereby ensuring that a meaningful biological signal is present in the data and that interpretation of the classification behavior is justified. Based on this approach, we were able to show that CLL, and to a lesser extent DLBCL, exhibit diagnosis-specific changes, whereas FL, MCcHL, and NScHL do not. We have revised the manuscript accordingly to clarify this rationale and hope that these changes address your concerns.

b. Figure 3 included some features, but how did we arrive at that? Does it mean all those features were significant (whether positively or negatively)? It's unclear to me.

Answer: Thank you for your helpful feedback. We have revised the manuscript to clarify both the feature selection process and the conclusions drawn from it. To increase transparency, we restructured and refined Sections 2 and 3 of the Results and added further details to the Materials and Methods (see pages 5-10 and 13-18). We now clearly indicate the data sources underlying each analysis: in Table 1/Figure 2 (former Figure 3), the morphological features were derived from a computer vision–based pipeline using Imaris, while the distribution parameters were calculated through cell graph analysis with NetworkX. All colored results for a given diagnosis are significantly different from LA, and the direction of the effect (larger or smaller) was determined by comparing the medians. We hope these revisions have improved clarity and resolved the ambiguity.

c. I'm not sure if the analysis was done using deep learning based on 3D images, or the dataset has been reduced from 3D images to measurements outlined table 1. It goes back to how to understand the result in Figure 2.2 (which shows that the classification isn't good at all, but I'm not sure if that is the focus of the paper)

Answer: Thank you for pointing out the ambiguities in our description of the machine learning approach. In our analysis, we trained a multiclass bagged decision tree classifier based on the extracted features per image (pathomic profiles), i.e., the measurements.

We agree that the overall classification rates are not particularly high; however, this was neither expected nor the primary focus of our study. Our goal in the training phase was to achieve performance above chance, thus enabling a meaningful biological interpretation of the classification results. The good multiclass classification performance for CLL and DLBCL demonstrates diagnosis-specific pathomic changes in the TAMs. The lower classification performance for NScHL, MCcHL, FL, and LA suggests that these entities do not exhibit clear differences from reactive states or from the other neoplastic conditions. We have revised the manuscript to clarify these points and avoid potential ambiguities.

d. It'll be good to have some literature research and outline what is unique about the study. Is it the data? Or the method? or the finding?

Answer: Thank you for pointing out that the uniqueness of our study was not fully explained. Previous literature has described functional differences between TAMs and reactive macrophages, as well as their general tumor-promoting role. Other studies have shown that increased macrophage/TAM density is often associated with poor prognosis. However, these functional descriptions are rarely diagnosis-specific, and even when density has been examined in individual diagnoses, it represents only a superficial measure of pathomic changes during tumorigenesis. To address this gap, we combined state-of-the-art digital pathology methods with a unique 3D dataset to characterize both morphological and distributional changes. In this way, our work provides a tumor-specific and detailed characterization of TAM alterations, links them to potential functional implications, and establishes a foundation for more comprehensive research on TAMs in the context of tumorigenesis. We have emphasized these aspects more clearly in the revised Introduction and Discussion to better convey the scientific added value of our study.

3. Reviewer #3:

a. Introduction, The background provided in the manuscript is incomplete and lacks clarity. For example, the authors must clearly explain why it is important to study these two apparent types of tumor-associated macrophages. What roles do they play in tumors? What markers (e.g., CD68+ or CD163+) identify each type of macrophage? Unfortunately, this critical information is missing. The authors wrote as though the audience were pathologists already familiar with the importance of their work. While a comprehensive review of the field is not expected, the manuscript should at least explain the role of these markers, the functions of the macrophages in malignant tissues, and the significance of distinguishing between reactive and neoplastic lymph nodes.

Answer: Thank you for this valuable comment. We have revised the introduction to provide a clearer and more comprehensive background on the role of macrophages in the (tumor) microenvironment, with particular attention to their functional heterogeneity. In general, M1-polarized macrophages are pro-inflammatory and have antitumor effects, whereas M2-polarized macrophages promote tumor growth, angiogenesis, and immunosuppression. Immunohistochemically, CD68 is frequently used as a general macrophage marker and, in many studies, as a proxy for M1-like macrophages. CD163 is frequently used to identify M2-like macrophages. However, TAMs often display mixed or intermediate phenotypes that cannot be clearly assigned to macrophage classes. Furthermore, CD163-positive macrophages have been primarily studied in the literature and established as tumor drivers, but the role of CD68-positive macrophages in tumor biology is less well understood. To provide a more comprehensive picture, we decided to examine both CD68- and CD163-positive macrophages. By explaining these aspects in more detail in the revised introduction, we aim to clarify the rationale and significance of our study for both pathologists and a broader audience (see pages 3-5).

b. Methodology, Although the authors provide a detailed description of tissue preparation, the methodology related to the central objective of the study is poorly addressed. If the study focuses on the morphology of different macrophage types, the introduction should highlight any prior associations between macrophage morphology and pathological states (e.g., benign, malignant, or reactive).

Answer: We appreciate this helpful comment and have revised the introduction accordingly. Previous studies have shown that functional differences in immune cells are often reflected in altered morphology. In the case of macrophages, this is exemplified by tingible body macrophages in germinal centers of lymph nodes, which are characterized by pronounced phagocytic activity rather than classic pro- or anti-inflammatory functions. These macrophages display a markedly larger and rounder morphology. Since functional differences between TAMs and reactive macrophages have been described, it is reasonable to hypothesize that they also differ morphologically. To our knowledge, however, direct morphological studies on TAMs have not yet been conducted; existing research has primarily focused on macrophage density within the tumor microenvironment. Our study was therefore designed to address this gap.

c. Additionally, questions such as whether reactive nodes are exclusively present in cancer and the importance of studying the distribution of tumor-associated macrophages within nodules remain unanswered. This contextual background is essential for interpreting Table 1.

Answer: Thank you for this insightful comment. Lymph nodes are a fundamental component of the immune system and are present in every individual, independent of cancer status. They are biopsied not only in cases of confirmed malignancy but also during the diagnostic examination of persistently enlarged nodes. Such enlargements may also result from reactive lymphadenopathies rather than neoplastic disease, as was the case in our cohort with diagnosed lymphadenitis. These patients exhibited inflammatory but purely reactive changes without evidence of malignancy and therefore served as an appropriate control group.

This control group enabled us to investigate pathomic differences of TAMs between different neoplastic entities and in comparison to reactive macrophages. In our study, these differences included both morphological and distributional characteristics. We considered distributional aspects because macrophage activity can also be reflected by patterns of accumulation. Under reactive conditions, for example, macrophages are known to cluster in the marginal sinus near afferent lymphatics, forming an effective first immune barrier. In contrast, tumor microenvironments such as Hodgkin lymphoma show enhanced macrophage–tumor cell interactions and higher macrophage densities. To capture such patterns, we applied a cell graph approach to assess local clustering tendencies. These points have now been emphasized more clearly in the introduction and discussion to strengthen the rationale and context of our work (see pages 10-12).

d. The authors mention in section 4.4 (lines 236-237) that they studied 24 features "for each staining individually." However, this statement is ambiguous. Did they analyze all the samples they had? How did they determine specificity? The manuscript does not clarify whether a specificity and sensitivity analysis was performed. It is well known that when studying pathological or immunohistochemical tissue samples, multiple images of the same tissue must be analyzed to ensure accuracy. A diagnosis or feature cannot be determined from a single image. This highlights a potential area for improvement using digital methods or AI in pathology.

Answer: We apologize for the ambiguity of our original description. In the revised manuscript, we have refined and expanded the sections on data acquisition (feature extraction) and data analysis (feature evaluation) to improve clarity and reproducibility. We now explicitly discuss the influence of image selection, image size, and the number of sections analyzed (see pages 15-18).

It is well established that, especially in diagnostics, multiple sections per patient are required. However, providing a diagnostic tool was not the goal of this work. Our focus was to investigate pathomic changes in macrophages across different reactive and neoplastic entities and to discuss their possible functional implications. For this purpose, we trained a bagged decision tree classifier. To ensure consistent case representation and

avoid overrepresentation, we included only one image section per case in each training cycle.

To evaluate classification performance per diagnostic entity, we examined accuracy across different training and test data splits, which we considered sufficient for our study objectives. In addition, in response to your valuable suggestion, we performed a one-versus-rest analysis and included the corresponding ROC curves in the appendix. We hope this increases the transparency and robustness of our analysis.

e. The authors state in the results (line 121) and methods (lines 240-241) that they analyzed a single selected image per case 1,000 times. However, they do not explain how these images were selected, nor do they address potential biases in this selection process.

Answer: Thank you for pointing out this potential source of confusion. To prevent overrepresentation, we reduced each case to a single image and, for consistency, always selected the first image listed in the underlying table of pathomic profiles. The reduced dataset was then split 1,000 times into different training and test sets, on which we trained the model and assessed classification accuracy. Repetition of this procedure minimized the influence of individual cases on performance evaluation. We acknowledge, however, that reliance on a single section per case may introduce bias, and we now explicitly discuss this limitation in the revised discussion (ll. 244-246).

f. Results, The authors fail to adequately describe how they constructed the pathomic profile, which appears to be critical for interpreting their results. They vaguely mention a "hybrid approach" (lines 168-169) but do not explain how Figure 2 was created or how the specificity percentages shown in the figure were calculated. Similarly, they present a figure correlating the pathomic profile with pathological diagnoses but do not describe the process for constructing this profile.

Answer: Thank you for pointing out these ambiguities. We have thoroughly revised and expanded the Methods section to provide a detailed description of the creation of the pathomic profile, including the definition of the "hybrid approach" and the generation of Figures 2 and 3 (see pages 8-9). To enhance transparency, we have also added process diagrams that illustrate the data extraction steps, the data processing steps, and the underlying datasets (see pages 15-17). Furthermore, we have restructured the Results section to present the workflow and outcomes more clearly, thereby facilitating a better interpretation and understanding (see pages 5-10). We believe these revisions resolve the ambiguities and make the methodology more transparent and reproducible.

g. In addition, they claim to have calculated specificity using the Mann-Whitney U test. Why didn't they use ROC curves, which are standard for evaluating specificity and sensitivity? Perhaps they lacked quantitative data, but the manuscript does not provide enough detail to ascertain this.

Answer: Thank you for pointing out the source of this confusion. For the prototyping shown in Figure 2 (former Figure 3), we used the Mann–Whitney U test to assess individual parameters for significant differences between reactive tissue and neoplastic entities, with significant results highlighted in color in the corresponding table. To evaluate classification performance, we calculated the accuracy for each entity individually.

We initially chose not to include ROC curves, as they are primarily suited for binary classification and provide only limited insight in a multiclass setting. ROC curves do not adequately reflect misclassification patterns and overlaps between diagnostic groups, which were central to our analysis of diagnostic comparability.

Nevertheless, in recognition of the general value of ROC curves for assessing classifier performance, we have now included a one-vs-rest ROC analysis for the multiclass scenario in the appendix. We hope that these additions, together with the revised and more clearly structured Results section, have resolved the ambiguities.

h. The discussion is weak, as it relies on flawed methodology and incomplete results. Furthermore, the manuscript lacks a clear conclusion. The authors' statement in lines 184-187, suggesting that pathomic properties can be integrated into histopathological assessments to enhance functional descriptions of neoplastic aberrations, cannot be substantiated based on the presented data

Answer: Thank you for your thoughtful evaluation. We have carefully revised the Discussion to strengthen its focus and provide clearer conclusions. Specifically, we removed overstatements that were not directly supported by our data (e.g., the claim that pathomic properties can already be integrated into routine histopathological assessment). Instead, we now explicitly emphasize the methodological contribution of our study and highlight that our findings establish a diagnosis-specific 3D baseline of TAM characteristics, which can serve as a foundation for future functional investigations. We believe these revisions address your concerns regarding methodology, interpretation, and the lack of a clear conclusion (see pages 10-12).

4. Reviewer #4:
   a. Enhance Visualization: Provide more detailed and intuitive visual representations (e.g., 3D interactive models or simplified graphs) to support the findings.

Answer: Thank you for this helpful suggestion. To enhance the clarity and accessibility of our results, we have added additional visualizations (new Figures 1 and 2, see pages 6-8), including 3D representations, and created workflow diagrams to better illustrate both the data extraction and the analysis workflow (new Figures 6 and 7, see pages 15-17). We believe these additions make the presentation of our findings more intuitive and easier to follow.

b. Strengthen Discussion: Compare findings with similar studies in greater depth to highlight the novelty and broader implications of the work.

Answer: Thank you for this valuable suggestion. We have substantially revised the Discussion to include a deeper comparison with related studies. In particular, we now contrast our findings with previous functional analyses of TAMs and highlight how our 3D pathomic approach extends beyond these methods. We also discuss relevant reports on TAM morphology and functional diversity, thereby underscoring both the novelty of our approach and its broader implications for understanding macrophages in the context of lymphoma biology. We believe these additions strengthen the discussion and better situate our results within the existing literature (see pages 10-12).

c. Supplementary Material: Add more detailed protocols or supplementary figures to aid reproducibility

Answer: Thank you for this helpful suggestion. To improve transparency and reproducibility, we have added new pipeline diagrams to the main text and included additional supplementary material. Specifically, we now provide p-values (Appendix, Figure 2) and supplementary plots like ROC curves. We hope this meets your expectations.
