# Supplementary material for: The three-dimensional landscape of tumor-associated macrophages in reactive and neoplastic human lymph nodes
Source: PLOS Digit Health. 2026 Feb 13;5(2):e0001227. doi: 10.1371/journal.pdig.0001227 (PMC12904395; doi:10.1371/journal.pdig.0001227)
Supplement: Attachment — Submitted filename: Response to Reviewers.docx [file pdig.0001227.s006.docx]

Response to Reviewers

Dear Leo Anthony Celi, dear Iqram Hussain,

We sincerely appreciate the time and effort you invested in evaluating our manuscript. We are grateful for your constructive comments and insightful suggestions, which have helped us substantially improve the clarity and robustness of our work. Below, we provide detailed point-by-point responses outlining how each comment has been addressed. We believe that the revisions made in response to your feedback have strengthened the manuscript, and we hope that it now meets the standards for publication in PLOS Digital Health.

Response to the suggestions raised by the editor:

1) I recommend including ROC-based evaluation, such as one-vs-rest ROC curves for each diagnostic entity, to demonstrate the classifier’s performance.

Thank you for this valuable suggestion. In response, we performed a one-vs-rest ROC analysis for each diagnostic entity across 1,000 stratified train–test iterations. The resulting averaged ROC curves and mean AUC values are now presented in Figure 8A. We have also added a detailed description of this analysis to Subsection 4.4 (“Data science”) of the Methods (p. 19, ll. 357-360).

2) A feature-importance analysis-e.g., shapley value or SHAP values, would help clarify which pathomic features most strongly influence the bagged decision tree predictions.

Thank you for this helpful suggestion. We conducted a feature-importance analysis using SHAP (SHapley Additive exPlanations) values computed from the classifier trained on the full dataset (without train–test splitting). The results are now visualised in Figure 8B. A description of this analysis has been added to Section 4.4 (“Data science”) of the Methods (p. 19, ll. 360-365).

3) Figure 7 should be improved with suggested recommedations and detials modeling information.

Thank you for this comment. We incorporated the requested modelling information into the newly introduced Figure 8, which presents both the ROC-based performance evaluation (Figure 8A) and the SHAP feature-importance analysis (Figure 8B) (p. 18/19, ll. 346-355). We hope that these additions directly address the recommendations while preserving the conceptual function and structure of Figure 7 within the manuscript.
